# More efficient reproducible research in hydrology: moving research down the academic career scale (MRDTACS)

**DOI:** 10.1098/rsta.2024.0296

**Published:** 2025-07-31

**Authors:** Rolf Hut, Caitlyn Hall

**Affiliations:** ^1^ Department of Water Resources, Delft University of Technology Faculty of Civil Engineering and Geosciences, Delft, Zuid-Holland, The Netherlands; ^2^ Biosystems Engineering Department, The University of Arizona College of Agriculture and Life Sciences, Tucson, AZ, USA

**Keywords:** hydrology, open science, FAIR science

## Abstract

Hydrology faces critical challenges in reproducibility, accessibility and collaboration, limiting progress and innovation. This article introduces ‘*moving research down the academic career scale’* (MRDTACS): the idea that work should be reproducible by someone at an earlier career stage and in less time than the original work. We advocate for research tools and methods to be accessible to students and early career researchers. By embedding open and findable, accessible, interoperable, reusable (FAIR) principles, modular tool design and user-friendly interfaces, we can lower barriers to reproducibility and foster equitable participation in hydrological research. Herein, we highlight practical strategies to empower researchers at all levels to build on existing work, reducing time spent overcoming technical challenges and enabling a deeper focus on innovation. When existing technologies and tools do not meet hydrology’s advancing needs and innovation is needed, we use eWaterCycle to illustrate how we have practically implemented open and FAIR principles to support MRDTACS. This approach advances equity and inclusivity while strengthening collaboration across academic and professional communities. By prioritizing reproducibility and transparency, we can create a more resilient and effective hydrological science field equipped to tackle urgent global challenges.

This article is part of the Royal Society Science+ meeting issue ‘Hydrology in the 21st century: challenges in science, to policy and practice’.

## 1. Introduction

At the 21st Century Hydrology meeting at the Royal Society in London, organizer Professor Hayley Fowler, in her opening presentation, highlighted the limitations in existing hydrological and atmospheric models, emphasizing the urgent need to address uncertainties (e.g. [1,2]) in our predictive systems. Tackling these uncertainties requires a deep understanding of the foundational scientific work and methods upon which current research is built or seeks to improve. Researchers often accept previous work published in academic literature at face value, but this becomes problematic when the assumptions and limitations underlying the conclusions are not fully considered [3,4]. Many assumptions are hidden within the model or analysis code and need to be clearly communicated in scientific publications. Hutton *et al* wondered: ‘Most computational hydrology is not reproducible, so is it really science?’ [5]. This is evident in a study by Stagge *et al*. [6], which found that only 1.6% of tested articles were reproducible. Opaque methodologies, tools and approaches contribute to a need for more transparency in published results and conclusions [6,7].

More concerning is that conclusions may not be reproducible—whether due to false positives or inadvertently building on research with subtle flaws not caught during peer review (e.g. [8]). When researchers attempt to build on incomplete or flawed work, or when research is not openly accessible, they are forced to start from scratch to verify the original findings. This wastes precious research time and thus hinders scientific progress. We must confront a critical challenge to further improve our science: current academic practices often make it difficult to reproduce and advance existing work effectively. This underscores two key reasons why ensuring reproducibility is essential. First, reproducibility allows for easier verification of research accuracy. Second, it enables researchers to build on prior work without replicating it. However, if reproducing work requires as much time and effort as the original research, the opportunity for efficient scientific advancement is lost.

Hydrological sciences have made significant strides towards improved reproducibility in the past decade. Many academic journals now require authors to make the data and code underlying their results publicly available or provide a clear justification if sharing is restricted due to legal, ethical or other constraints ([9–11]). The days of ‘email the author (who may have left academia) to request data’ should largely be left behind.

However, a closer look reveals that even award-winning studies sometimes struggle to meet full journal standards for reproducibility. The 2022 and 2023 Dooge Award winning papers in *Hydrology and Earth System Sciences* highlight progress and inconsistencies. While the model code is provided in Morin *et al*. [12], the specific scripts needed to run the model and replicate exact results still need to be included. Similarly, Mai *et al*. [13] provide scripts to generate figures from model output data in large intercomparison studies, but they need to include scripts to run the models themselves. Peters *et al*. [14] neither shared code nor provided data openly, opting instead to make data available on ‘reasonable request’.

These inconsistencies reflect a broader issue: achieving accurate, comprehensive reproducibility requires substantial time and effort. If reproducing the work by another scientist takes as much time as it cost the original scientist to produce the work, then we cannot effectively build on each other’s work. A reproduction, including learning all the knowledge needed to execute and understanding the work, should take less time than the original research did. On a career level, this leaves new scientists with room (time) to improve on the existing work and bring science further. Hydrologists work in many sectors, including academia, consultancy and government. Academic hydrological research stands apart from those that actively do it: BSc, MSc, PhD students and post-docs typically quickly move on after their thesis/project finishes. For them, investing in making work reproducible is even more dis-incentivized compared to other sectors!

**Figure 1.**
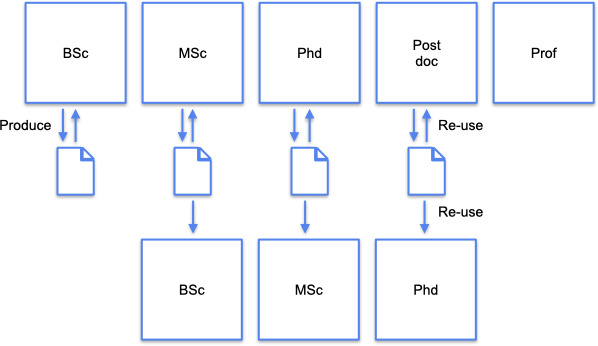
A graphical representation of ‘moving research down the academic career scale’ (MRDTACS). We argue that work produced at some stage of the academic career should not only be reproducible by peers on the same tier of the career but also by people on an earlier tier. This requires taking into account educational aspects when making a work reproducible. See the electronic supplementary material for additional information on the role of the ‘prof’ in this framework.

This article introduces the idea of deliberately ‘moving research *down the academic career scale’* (MRDTACS). Academic research should not just be reproducible but reproducible by individuals at earlier stages of their academic careers and in less time than the original researchers. See figure 1 for a graphical overview of this idea. This implies that research tools and methods should be accessible to everyone, from senior researchers to students beginning their academic journeys. The onus of facilitating MRDTACS is on people who build tools that academic hydrologists use and on people who supervise the ones doing the actual research (i.e. the onus is on academic professors). Achieving this will enable early career researchers to effectively build on the work of their more senior peers and keep the progress of science going.

Implementing open and findable, accessible, interoperable, reusable (FAIR) principles with intentionality around MRDTACS can make research more reproducible and actionable, directly addressing current models’ uncertainties (and many other challenges in hydrology), as Professor Fowler emphasized. This article discusses achieving MRDTACS from technical and educational perspectives, focusing on undergraduate and graduate academic contexts.

While MRDTACS is initially framed through an academic lens, its foundational goal—making research more reproducible and transferable—extends well beyond academia. By prioritizing MRDTACS, we aim to ensure that hydrologists with academic training are better prepared to develop new tools and apply translational skills across sectors. Hydrologists increasingly work in interdisciplinary, governmental, non-profit and industry settings, where they both generate and apply research outputs. Advancing FAIR principles across the hydrological community requires not only supporting students and early career researchers but also enabling academic development for senior professionals, fostering cross-disciplinary collaboration and ensuring applied practitioners can access, understand and adapt research. In this article, we introduce MRDTACS in the academic context. By adopting this during the training stage of future hydrologists it will ultimately reach across the full spectrum of hydrological science—regardless of discipline, sector or career stage.

Many of the strategies outlined build upon our previous work, *A Hydrologist’s Guide to Open Science* [10], which addresses the practical, institutional and cultural challenges that make reproducible hydrology difficult to achieve. In that work, we highlighted time constraints, uneven access to training, and the lack of credit systems for open and reproducible practices as persistent barriers. We also acknowledged that reproducibility takes significant time and effort—sometimes rivalling the effort required for the original research—and that these efforts are often deprioritized without structural support. Building on that foundation, this article specifically centres on enabling MRDTACS, offering practical strategies to reduce friction at the point of use: for example, by encouraging modular workflows, leveraging community-built platforms and designing teaching materials that reinforce reproducible practices early on. We do not claim that these strategies resolve all barriers—especially those related to funding, evolving software dependencies or long-term maintenance—but rather that they are a step towards reducing inequities in who can build on existing research. We use our work on the eWaterCycle project as an illustrative case study to demonstrate how platforms and educational design can support MRDTACS and reduce technical overhead. We believe reproducibility should be treated as a shared responsibility between individual researchers, institutions and funding bodies, and that adopting reproducibility principles will be most effective when embedded within broader systemic change.

To advance science, the next generation of researchers must be equipped to build on existing research without retracing every step (and misstep) we took. This requires not only accessible tools but also deliberate training in their use from the outset. By embedding these practices in technical workflows and education, we can empower future researchers to contribute effectively and confidently to hydrological science.

## 2. Turning MRDTACS into practice

Reproducibility in hydrological research is often hindered by common challenges. Below, we outline these challenges and propose solutions aligned with the principles of MRDTACS. Importantly, researchers do not always need to implement these solutions independently. Many academics, including our team, have developed platforms designed to address these issues directly, embedding solutions into their design. Our primary recommendation is to avoid ‘reinventing the (water) wheel’ [15] by utilizing existing open platforms whenever possible.

These platforms are specifically designed to maximize reproducibility by providing access to standardized research environments. They allow peers to run analyses, view results and make adjustments, creating collaborative spaces that support transparent and shared scientific inquiry. By enabling others to interact with your work, these platforms establish a foundation for rigorous, reproducible research and promote effective scientific collaboration.

Over the past several years, several platforms have emerged to support reproducibility in hydrology. Table 1 lists several of these platforms, detailing their specific strengths and challenges in the context of hydrological research. These tools enable researchers to perform analyses, store data and share code and results, with each platform tailored to different research needs. By fostering open and FAIR science, these platforms prepare future hydrology researchers to engage in collaborative and reproducible research environments.

**Table 1.** An overview of existing platforms and software tools that incorporate parts or all of the advice given in §2. The strengths and challenges of the different platforms are briefly summarized.

platforms	description/purpose	strengths	challenges
eWatercycle [16]	a platform supporting reproducible hydrological modelling and simulations for collaborative research	— provides access to diverse hydrological models, enhancing reproducibility — promotes cross-disciplinary collaboration	— requires advanced knowledge of hydrological modelling, which may be a barrier for beginners — initial set-up and high computational needs can be a constraint
HydroShare [17,18]	a platform supporting reproducible hydrological modelling and simulations for collaborative research	— enables seamless sharing of data and models, facilitating collaborative research — supports FAIR principles, improving accessibility and interoperability	— requires data management preparation to ensure effective sharing — integration with non-hydrology tools may be limited, potentially complicating workflows
PAVICS-Hydro [19]	designed for climate and hydrological modelling, providing tools for environmental data analysis	— integrates climate and hydrology models, ideal for climate impact assessments — user-friendly web interface simplifies complex analysis tasks	— offers limited customization, which may restrict advanced users — high computational demands can limit accessibility for smaller research teams
Binder (https://mybinder.org/) [20]	an open-source platform that creates and shares interactive research environments with ease	— simplifies sharing of code and analyses in an interactive format — compatible with Jupyter notebooks, widely used in hydrological research	— Public Binder instances are limited to simpler models due to resource constraints — not ideal for large datasets or resource-heavy hydrological models
Google Colab (https://colab.research.google.com/) [21]	a cloud-based platform for coding, primarily in Python, supporting data analysis and machine learning	— accessible for beginners and useful for testing basic models — built-in GPUs support faster computations for large datasets	— limited compatibility with certain hydrological software and models — memory and runtime limitations restrict large-scale simulations

Platforms like Google Colab and HydroShare offer accessible solutions for teaching and collaboration, standardizing environments and reducing compatibility issues. At more advanced levels, platforms such as eWaterCycle, PAVICS-Hydro and Binder allow students and researchers to engage directly with complex models, promoting open science principles and preparing them for a reproducible research ecosystem.

Many, including us, have argued that implementing reproducible research will improve the quality of our science [10,22,23]. We support the idea that future hydrology researchers gain essential skills in conducting rigorous, reproducible research and contributing to a more interconnected and resilient scientific community through these tools. By embedding platforms that promote transparency and collaboration into their workflow, researchers at all levels are better equipped to build on each other’s work, reducing the need to reinvent foundational processes and instead focusing on advancing new questions and solutions. This approach fosters a research ecosystem where collective knowledge grows more efficiently and findings are readily validated and extended.

For many hydrologists—particularly those trained in field-based or empirical methods—learning to use reproducible platforms can be a steep learning curve. To move research effectively down the academic career scale, we must scaffold these skills early and often. Training materials and platform design should be accessible to researchers from diverse technical backgrounds, ensuring students can confidently engage with and adapt existing tools. Embedding these practices in education prepares students to build on prior work and carry reproducibility forward into both research and applied hydrological practice. As we adopt and integrate these approaches into our teaching and mentorship, there are several critical questions to consider:

(1) What are the primary learning goals for my students? Suppose the objective is to understand modelling principles. In that case, providing students access to existing models and data within a streamlined environment allows them to focus on the science rather than the technical set-up.
(2) What support do students need to reproduce advanced research effectively? If more complex tools are reproducible, students can engage with professional models and data without struggling with compatibility or set-up issues.
(3) What challenges should be removed to keep students focused on learning? For example, if setting up a cloud-based analysis took months, providing students with an accessible set-up keeps them focused on core learning objectives.
(4) How can students build on this work to deepen their understanding and contribute meaningfully to research? Allowing students to explore and adapt work encourages both skill-building and research innovation.
(5) Am I prioritizing students’ learning over the advancement of my own research? Ensure that any research benefits are secondary to student education, respect students’ time and centre their development.

To prepare the next generation of hydrologists to innovate, we must equip them to use tools and understand their structure. Moving research ‘down the academic career scale’ means that even early career researchers should feel empowered to adapt and build on prior work. This requires transparency, robust documentation and interoperability—principles that make research reproducible while pushing scientific boundaries. By embedding these principles in technical research and education, we ensure that researchers at all stages are equipped to drive hydrology towards a more collaborative and transformative future.

The remainder of this section discusses common problems encountered when making one’s work reproducible and the solutions that we advise in light of MRDTACS. Note again that many platforms discussed above have already implemented these solutions.

### (a) Problem: software dependencies

No model or code exists in isolation—managing software is essential. Hydrological research software often relies on specific versions of programming languages and various libraries, both native and third party as we and others have indicated [5,15,24]. For accurate reproduction of results, researchers need to match these exact versions; even minor mismatches can affect model outputs. The following solutions offer a scaffolded approach—from simple to advanced—for effectively handling dependencies in hydrological modelling, ensuring consistent and reproducible research outcomes across different set-ups.

Teaching dependency management to early career researchers and students is crucial for fostering a culture of reproducibility and rigour in hydrological modelling. When students learn to handle software dependencies effectively, they gain essential skills for setting up, running and reproducing complex models—foundational for credible research. By integrating dependency management into educational curricula, we prepare new researchers to engage with established research confidently, collaborate across diverse technical environments, and share their work in a way others can easily replicate. This hands-on experience builds technical competence and empowers students to contribute to a more collaborative, reproducible research community where scientific progress can build seamlessly from generation to generation.

#### (i) Solution: use software environments

A software environment is a cohesive set-up that includes analysis scripts, libraries and the necessary programming language(s) to perform one or multiple related tasks. This environment is internally consistent: if an analysis script requires a specific version of Python’s numpy library, that exact version of numpy is included in the environment, ensuring all dependencies align for accurate and reproducible results. In academic research, environments often serve a single researcher working within a specific field; for example, one environment might be dedicated to sensor projects, while another is configured for global hydrological modelling.

Modern tools like Anaconda and virtual environments in Python make switching between environments straightforward and allow users to export entire environments for others to replicate. Sharing research results alongside the code and environment generated significantly facilitates reproducibility, enabling others to build on the work more efficiently.

For intermediate students and researchers, learning to create and manage software environments (e.g. using Anaconda or virtual environments) is crucial. By guiding students through setting up, exporting and sharing their own environments, educators can instil best practices for reproducibility. Assignments that involve creating and managing environments, coupled with lessons on their role in research, help reinforce the importance of these skills in collaborative and transparent research.

### (b) Problem: unsupported software (post-doc-ware)

A quick chatGPT query yields a list of at least 18 different Python libraries that can read comma separated value (CSV) files. While it might be tempting to create a custom solution, chances are one of these libraries will meet your project’s needs. In our experience as teachers of future hydrologists, we have encountered many moments when those under our supervision tried to recreate the functionality of existing libraries, either because they were not aware of the existence of those libraries or because the functionality did not completely match their research requirements. Developing a custom parser may seem appealing for control, but unless you are prepared to maintain it across programming language updates, it can contribute to the dependency issues that hinder reproducibility.

#### (i) Solution: use existing libraries

By opting for well-documented, commonly used libraries and adjusting workflows to integrate them, you streamline your research and make it easier for others to build upon.

For example, using a widely supported library like Pandas instead of creating a custom CSV parser keeps dependencies stable and predictable. Adding comments to specify the library version and its source also improves reproducibility. Managing dependencies for larger projects becomes even more straightforward with environments that encapsulate all library versions and ensure consistency across set-ups, promoting collaborative compatibility.

Embedding these reproducibility practices—like using established libraries, documenting dependencies and sharing workflows—into educational curricula prepares students and early career researchers to build on existing work with minimal set-up challenges. This approach ensures that knowledge flows seamlessly to each new generation, fostering a research community focused on rigorous, accessible and collaborative science.

### (c) Hardware and computational resource limitations

Hydrological modelling performance is significantly influenced by the hardware on which it runs, and differences across researchers’ hardware set-ups—such as CPU core count, memory and chipset specifics, like how random number generation is handled—can affect model outputs, particularly as models become more complex. In addition, differences in file systems, including unique mounting points and directory structures, often cause workflows to break when transferred to other machines. Even if software dependencies are managed, it usually takes considerable time to adapt an analysis from one machine to another. This issue can significantly affect researchers with limited access to high-performance computing, creating inequities in who can successfully reproduce or expand upon complex analyses in hydrology.

Overcoming these technical barriers is essential for promoting equity in hydrological research. Researchers have varying levels of access to high-performance computing resources, and disparities in hardware can limit who can successfully reproduce or build on existing work. Addressing these challenges allows researchers from diverse backgrounds and resource environments to participate, ensuring that early career researchers and those with limited resources can contribute to and advance the field. By creating adaptable, hardware-agnostic solutions, we can support the next generation of hydrologists in accessing, reproducing and expanding on established research, promoting a more inclusive and collaborative research community.

#### (i) Solution: use containers for interoperability

Containers, such as those created with Docker, Singularity, Apptainer or Podman, offer a robust solution by providing a portable, standardized environment for research software. Containers encapsulate all dependencies, allowing code to run consistently across different hardware set-ups as long as a compatible container engine is present. Although setting up a container requires more initial effort than a simple software environment, a well-constructed container vastly improves the transferability and consistency of research software across systems. For example, containers enable researchers to bypass specific hardware constraints, ensuring that code and models perform identically on any compatible system.

Teaching students and early career researchers to create and use containers provides them with practical skills in managing diverse computational environments. This hands-on experience prepares students to tackle real-world research challenges, where adapting workflows across different systems is often necessary. By learning containerization, we hypothesize that students gain resilience in handling IT challenges, promoting a more equitable research environment where computational limitations are minimized and workflows are portable and accessible across diverse settings.

#### (ii) Solution: provide scaled-down data for accessibility

Large-scale studies that require extensive computational resources can be challenging to reproduce. By providing a smaller ‘cut-out’ of the dataset, such as a limited geographic region or a shorter timeframe, researchers enable others to test the methodology without needing access to the same level of resources. For example, sharing a smaller version allows others to understand and replicate core methods if a study requires a full month of supercomputer time to analyse multi-region or multi-model comparisons. For example, Aerts *et al.* [25] studied hundreds of catchments of the CAMELS dataset and provided Jupyter notebooks to analyse single catchments as electronic supplementary material [26]. This approach lets peer reviewers and collaborators validate a smaller portion of the analysis, increasing confidence in the findings.

Offering scaled-down versions of data makes complex studies more accessible for students and researchers from diverse backgrounds, including those with limited resources. Instructors can use these manageable datasets to introduce students to large-scale hydrological analyses without the high computational demands, allowing them to grasp the methodology and underlying concepts effectively. This practice prepares students to extend these methods to new contexts in their future work, bridging the gap between theory and application in hydrological modelling.

### (d) Problem: complex, multi-step workflows

Hydrological workflows often involve numerous, complex steps, from initial data processing to model calibration and scenario analysis. These multi-layered workflows can be too complex for others to easily understand and reproduce, particularly for students or early career researchers needing more familiarity with every stage of the process. However, while recreating an entire workflow may be impractical, smaller, well-defined components can still offer significant value to other researchers and students.

#### (i) Solution 1: modularize workflows for interoperability

Breaking down workflows into modular steps with logical save points can improve accessibility and reusability. Storing intermediate data in standardized formats at each save point allows others to engage with individual workflow components without rerunning the entire process. For instance, if a workflow includes global grid interpolation, sharing this as a standalone module enables other researchers to apply it to their work without requiring access to the full workflow. This modular approach promotes reproducibility and flexibility, as researchers can build on specific components that meet their unique research needs.

Teaching students to modularize workflows and create standardized save points instils essential skills for reproducibility and collaborative research. Knoben *et al*. [27] provide a great set of tools for this with CWARHM. By organizing workflows into accessible, independent modules, students learn to design research processes that others can easily understand and expand upon. This approach prepares students for future projects where modular, interoperable methods enhance collaborative potential and simplify adaptation to new applications.

#### (ii) Solution 2: publish custom tools as libraries

When certain workflow components are repeatedly used, consider publishing them as independent, reusable libraries. This not only makes the tools accessible to others but also ensures their long-term usability across various research contexts. For example, in our work with eWaterCycle, we created a command-line tool called ERA5CLI to streamline bulk downloads of climate data from the Copernicus Climate Data Store. By making ERA5CLI a standalone library, we ensured it would be valuable for researchers beyond our own platform [28]. Another example is the development of the re-gridding tool xarray-regrid as part of the excited project [29]

We believe that encouraging students to publish reusable tools fosters a culture of open science and community support. By contributing libraries to the broader research community, students learn the importance of developing software with wider applicability in mind. This practice builds technical skills and strengthens their role within the hydrological research ecosystem, preparing them to contribute effectively to collaborative and effective scientific work.

### (e) Problem: systemic barriers to FAIR in hydrology

While many reproducibility challenges are technical in nature, they are deeply intertwined with systemic barriers that affect how researchers—particularly students and early career scientists—are trained. Hydrologists often receive formal education in field methods, geospatial analysis or hydrologic theory, but may not be equipped with the software development, data management or workflow documentation skills needed for reproducible research. The 2023 UK Hydrology Skills Survey found that many professionals across academia, government and consulting lack confidence in scripting and software versioning—core competencies for reproducibility [30].

These skill gaps are compounded by broader academic structures. Faculty may lack time, support or incentives to teach open and reproducible science practices, particularly when these practices are not explicitly rewarded in hiring, publication or promotion. As highlighted in Hall *et al*. [10], these systemic disincentives can create a culture in which reproducibility is seen as aspirational but non-essential—especially under pressure from grant deliverables, short academic timelines and limited computational resources. Moreover, as software ecosystems evolve, even well-intentioned efforts to make work reproducible can fail without ongoing maintenance and institutional support [31,32].

#### (i) Embed reproducibility in pedagogy and shared infrastructure

To overcome these barriers, reproducibility must be embedded early in research training and reinforced through mentoring and curriculum design. MRDTACS offers a pedagogical lens. It encourages researchers to design their work so it is not only reproducible, but specifically reproducible by someone with less experience or technical training. In this way, reproducibility becomes a teachable and intentional outcome, not just a technical ideal. Students and early career researchers benefit when tools, workflows and documentation are designed to be approachable. Educators benefit when they can teach reproducibility through hands-on engagement with real research.

Platforms like eWaterCycle help by reducing technical overhead while maintaining transparency. These tools make it easier to integrate reproducibility into both classroom and research training settings. Instructors can focus on core hydrological concepts, while students gain practical experience with modular workflows, containerized environments and open data. This scaffolds both conceptual understanding and technical skills. When students learn reproducible research practices, they are better prepared to contribute to innovation—whether in academic research, interdisciplinary collaboration or applied hydrological work in government, industry or the non-profit sector. To support this, institutions and professional communities must recognize reproducibility as a core part of research excellence and provide the infrastructure and incentives needed to sustain it.

## 3. Developing platforms for hydrological research that MRDTACS: the eWaterCycle case example

When existing platforms, models or libraries fall short of meeting your research needs, developing your own tools provides an opportunity to advance hydrological research while fostering collaboration and accessibility. Following best practices, such as adopting open-source frameworks to encourage contributions and designing modular components for diverse contexts, can significantly reduce barriers to collaboration. Scaled-down versions and user-friendly interfaces help engage non-specialists, broadening participation across varying levels of technical expertise [33]. To ensure reproducibility and ease technical challenges, tools should utilize containers to standardize dependencies and include detailed documentation with versioning to track changes transparently. Rigorous testing across regions and scenarios is also essential to demonstrate adaptability and clarify any required modifications for different applications.

Multiple platforms have been developed, or are currently being developed, that integrate many of the solutions from §2, see the list provided in table 1 for an incomplete overview. While most of these platforms, ours included, have been primarily designed with research, not education, in mind, the innovations they bring do facilitate the practices of MRDTACS. In this opinion paper, where we base our opinion on the experiences of our own teaching and education, below we detail how the eWaterCycle platform implements the suggested solutions. Before adopting a new platform, we strongly advise looking at multiple platforms and matching which facilitates MRDTACS for your own education.

The eWaterCycle platform for open and FAIR hydrological modelling [16] exemplifies how hydrologists can collaboratively work with both large datasets and complex models while adhering to open science principles; eWaterCycle serves as a practical example of how strategic tool development can empower researchers, reduce technical barriers and foster innovation by minimizing the software challenges that often hinder hydrological research, enabling more time to be spent on advancing new ideas.

Central to eWaterCycle’s design is the separation of hydrological models from the experiments conducted with them. Models are presented to users as objects accessed through a common interface (BMI, [34]), and they run in containers to ensure consistency and portability across systems. This design enables models with entirely different programming languages and dependencies to function seamlessly within the platform. Since v. 2 (Hut *et al.* [16] describes v. 1), models have been treated as ‘plugins’, allowing model developers to maintain control over their codebase while making it easy for others to contribute new models to the platform.

**Figure 2.**
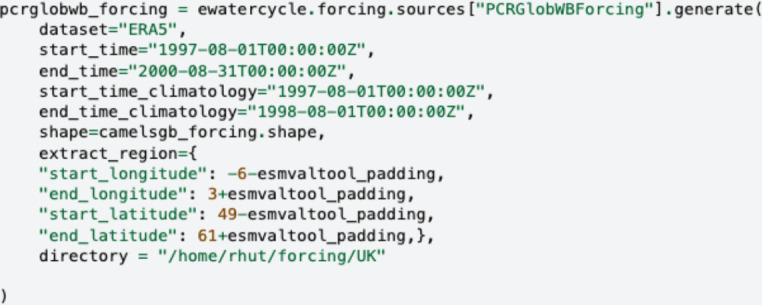
Example code from eWaterCycle for generating forcing (precipitation and temperature) data for the PCRGlobWB model. This shows that a user does need domain knowledge from hydrology, but does not need to know how to download the ERA5 source files and pre-process them to prepare them for PCRGlobWB, the platform handles this.

A guiding principle of eWaterCycle is that ‘you lose 90% of your potential users if they have to use the command line’. To honour this, the platform simplifies workflows by handling key hydrological actions at a high level. For instance, generating input files for the hydrological model PCRGlobWB [35] using CMIP climate model data for the SSP585 scenario requires only a single line of code (figure 2). This approach ensures that users need domain knowledge in hydrology while the platform abstracts away the technical complexities of downloading and processing large datasets, such as ERA5.

A key insight from eWaterCycle is that making research reproducible for students enhances their education and supports broader research applications. For example, suppose a climate change effect study on flood occurrence in the Congo Basin is reproducible. In that case, students can replicate the analysis in the Rhine or other watersheds, reinforcing their learning while extending the study’s applicability. To further this goal, we are actively developing open educational resources using eWaterCycle, based on teaching materials from Delft’s Environmental Engineering programme. This effort underscores the platform’s potential to democratize hydrological research and foster collaboration across academic levels.

By reducing technical barriers and promoting reproducibility, eWaterCycle exemplifies how tools can facilitate collaborative research, empower students and enable a more inclusive hydrological science community.

## 4. Essential takeaways for a collaborative and reproducible future in hydrology where research moves down the academic career scale

The future of hydrological research depends on practices that make research more reproducible, accessible and collaborative. As a closure to this article, here are four key takeaways to guide researchers, educators and tool developers towards creating a field that facilitates innovation and engagement at all academic levels.

### (a) Write for reproducibility, and give students the opportunity to demonstrate it

Ensure your research is documented enough for students and collaborators to reproduce it without ambiguity. By encouraging students to reproduce and extend their work, we shift away from ‘black box’ science and foster a deeper understanding of hydrological methods. Prioritizing transparency empowers the next generation of scientists to meaningfully engage with models and methods, creating a foundation for further innovation.

### (b) Pass on good research habits early

Good research practices develop through mentorship. Train students and early career researchers in essential technical and organizational skills—managing dependencies, modularizing workflows and thorough documentation. Teaching reproducibility and transparency from the outset builds a culture of open science, ensuring these principles become standards, not options. Using platforms that enforce or encourage these best practices makes this easier.

### (c) Leverage existing tools and innovate only when needed

Begin by assessing existing platforms, libraries and models that can support your research to avoid duplicating efforts. Using established tools saves time, promotes compatibility and enhances collaboration. If gaps remain, develop new tools with accessibility, modularity and adaptability in mind so others can readily understand, apply and extend.

### (d) Designing for interoperability and accessibility

Removing barriers such as hardware limitations and complex workflows makes research more inclusive and accessible across academic levels and career paths. Researchers working in applied settings, adjacent disciplines or outside academia entirely also benefit from reproducible tools and transparent methods. By designing platforms and workflows that are adaptable across computing environments and disciplinary contexts, we support a more collaborative and globally relevant hydrology community. MRDTACS is not just about mentoring the next generation of researchers, practitioners and decision-makers but also about making hydrology’s progress available to all who seek to apply or build upon it.

### (e) Supporting change for FAIR hydrology

To sustain MRDTACS, institutions must also shift how they value research contributions. Reproducibility efforts—including documentation, code sharing and database and tool maintenance—require time and are often invisible in traditional academic reward structures. Recognizing and crediting these practices in hiring, promotion and publication processes are essential to making reproducible hydrology the norm, not the exception [10].

Building hydrology research tools that facilitate collaboration and innovation across career stages and sectors creates pathways for students, early career researchers, applied practitioners and established scientists to work together more effectively. By embedding open, reproducible and inclusive research practices into both education and professional workflows, we can make hydrological science more accessible, equitable and actionable. This not only supports learning and innovation within academia but also strengthens the ability of hydrologists working in government, industry and non-profit roles to adapt and apply research in practice. MRDTACS provides a guiding lens for designing reproducibility with downstream users in mind—those with fewer technical resources, different disciplinary backgrounds or limited time. By prioritizing intentional reproducibility, we create space to focus less on overcoming technical barriers and more on growing the field, advancing scientific inquiry and delivering transformative, real-world solutions.

## Data Availability

Supplementary material is available online [36].
